# How Does Thyroid Hormone Profile Differ on and Off Replacement Treatment?

**DOI:** 10.1111/cen.15185

**Published:** 2024-12-19

**Authors:** Adrian H. Heald, Lakdasa D. Premawardhana, Peter N. Taylor, Adam Baker, Nadia Chaudhury, Anthony A. Fryer, Onyebuchi E. Okosieme, Colin M. Dayan, Mike Stedman

**Affiliations:** ^1^ The School of Medicine and Manchester Academic Health Sciences Centre University of Manchester Manchester UK; ^2^ Department of Endocrinology and Diabetes Salford Royal Hospital Salford UK; ^3^ Thyroid Research Group Systems Immunity Research Institute, Cardiff University School of Medicine Cardiff Wales UK; ^4^ Department of Endocrinology and Diabetes University Hospitals Coventry and Warwickshire Coventry UK; ^5^ Worcester College Oxford UK; ^6^ School of Medicine Keele University Staffordshire UK; ^7^ Res Consortium Andover UK

**Keywords:** thyroid hormone profile, treated hypothyroidism, untreated and euthyroid individuals

## Abstract

**Introduction:**

There continues to be much discussion around optimisation of thyroid hormone status in hypothyroid individuals. We here looked the way that free T4(FT4) and thyroid‐stimulating hormone (TSH) related to each other in a large laboratory sample of people who underwent a thyroid function test (TFT), split between those on levothyroxine replacement (monitoring test) and those who underwent a test to check for thyroid hormone imbalance (diagnostic test; not on levothyroxine).

**Methods:**

TFT test (FT4/TSH) results were extracted from the Salford Royal Hospital Laboratory Information Management System during 2009–2012. This was a single site study. Requests includes a tick box for ‘on levothyroxine’ (yes or no). To minimise comorbidity effects, only samples taken in General Practices were used. For untreated patients only those who had single tests results were used; for treated patients, the median value across all their results was used. Cluster analysis considered an ellipse with centre on median values for log (TSH) and FT4 and the vertex based on 5% and 95% percentile values of both. The percentage of patients falling outside the ellipse boundary was considered for both treated and untreated populations.

**Results:**

The total data set included 290,000 tests on 130,000 individuals. After filtering, FT4/TSH results were used from 12,006 (F 9231/M 2775; age < 60 5850/age ≥ 60 6567) treated patients with 43,846 test results. These were compared to the single results for 43,394 untreated patients (F 24,386/M19,008; age < 60 32,537/age ≥ 60 10,857). Cluster analysis showed for untreated patients, median values for TSH and FT4 were 1.8 mU/L and 15.5 pmol/L, respectively, with 24% of patient results falling outside the untreated 5%/95% percentiles. For treated patients, the median TSH was 2.3 mU/L (+30% vs. untreated) and FT4 was 18.9 pmol/L (+22% vs. untreated), with 22% of treated patients falling outside the treated 5%/95% percentiles. When considered against the untreated limits, 68% of treated results fell outside (split male 63%, female 70% and age < 60 67%, Age ≥ 60 64%).

**Conclusion:**

The current treatment regimens of either low or high dose levothyroxine are not delivering the expected laboratory TFT profiles, with significant numbers of treated patients being well outside the expected values: both TSH and FT4 being significantly higher. This effect appears to be more prevalent in women than men.

## 1 Introduction

Hypothyroidism is estimated to affect around 3% of the population in Europe [1], and is more common in certain groups such as females and older adults [2]. Clinically, hypothyroidism presents with symptoms such as cold intolerance, fatigue, and weight gain [3]. Biochemically, hypothyroidism is diagnosed through measurement of thyroid‐stimulating hormone (TSH) and free thyroxine (FT4), with increased TSH and decreased FT4 the typical pattern seen in patients with overt hypothyroidism. Additionally, there exists a subset of the population with high TSH levels but normal FT4 and no or minimal symptoms; this is known as subclinical hypothyroidism [4].

The most prescribed treatment for hypothyroidism is levothyroxine (LT4) monotherapy [5], with this medication being converted into the more metabolically active thyroid hormone, triiodothyronine (T3) [6], by deiodinases within the body. LT4 is a very widely prescribed drug, with 33.8 million prescriptions in the UK alone in 2022 [7]. Other thyroid hormone treatments, such as natural desiccated thyroid (NDT) and liothyronine (LT3) are available but are used much less commonly in modern practice [8].

The ideal therapeutic goal in hypothyroidism would be to restore clinical and biochemical euthyroidism via physiologic thyroid hormone replacement. This concept may seem straightforward, but there are subtleties that have only recently been recognised. In the majority of patients, LT4 treatment will both normalise TSH levels and lead to symptom resolution. However, it is estimated that around 5%–15% of patients taking LT4 still experience symptoms of hypothyroidism, even with normalised TSH levels [9, 10]. This may be due to LT4 monotherapy not restoring T3 levels to the normal range in a subset of patients [11, 12], perhaps due to polymorphisms within the genes which encode peripheral deiodinase enzymes [13].

Additionally, multiple studies [14, 15] suggest that as many as 40% of patients taking LT4 have TSH levels outside of the normal range, indicating under‐ or over‐replacement of thyroid hormones. Certain patient factors, such as sex, age, and duration of treatment, may influence this under‐ or over‐treatment [16]. Significantly, both under‐ and over‐treatment of hypothyroidism are associated with increased all‐cause mortality [17, 18].

This study aims to investigate further the differences in thyroid hormone levels and health outcomes in people on thyroid hormone replacement therapy compared with people being screened for thyroid disorder and not taking levothyroxine. Better understanding of the relation between thyroid hormone replacement therapy and actual levels of TSH, Free T3 (FT3), and FT4 in patients could allow more tailored and effective therapy for people with hypothyroidism.

## 2 Methods

Thyroid function test results (FT4 and TSH) were extracted from the Salford Royal Hospital (UK) Laboratory Information Management System for the period 1 January 2009–31 December 2012. This included tests conducted on request from both primary care (denoted by general practice code) and secondary care (denoted by hospital clinician and clinic location code). The clinician request includes a tick box for ‘on levothyroxine’ (yes or no). Age and sex of patients was also available. To minimise comorbidity effects, only samples taken in General Practices (not from secondary care) were used and for untreated individuals only those who had single tests results were used (screening test). For treated patients (on thyroid hormone replacement), the median value across all their results were used.

Assays for TSH and FT4 were undertaken on the Siemens Centaur (Farnborough, Hampshire). This was a single site study. The reference range for TSH was mu/L 0.35–5.50 and for FT4 was 10–20 pmol/L.

The data was fully anonymised before analysis. The project was approved by the Northern Care Alliance R&I committee (ref NCA 2023/24).

### 2.1 Statistics

Cluster analysis considered an ellipse with centre on median values for log (TSH) and FT4 and the vertex based on 5% and 95% percentile values of both. The % of patients falling outside the ellipse boundary was considered for both the treated and untreated population.

The development of median values FT4 and TSH and by nominal age was examined for both treated and untreated populations.

Analysis examined the impact of splitting the population by age (< 60 and ≥ 60) and sex on the % difference between median values of TSH and FT4.

## 3 Results

The complete data set included 290,000 tests for 130,000 patients. However, the FT4/TSH results were used from 12,006 (F 9231/M 2775 age < 60 years 5850/age ≥ 60 years 6567) * treated patients with 43,846 test results. These were compared to the single results for 43,394 untreated patients (F 24,386/M19,008 and age < 60 32,537/age ≥ 60 10,857). The age category total is greater than 12,006, because TFT in some people were checked and then subsequently rechecked on/after their 60th Birthday.

**Figure 1:**
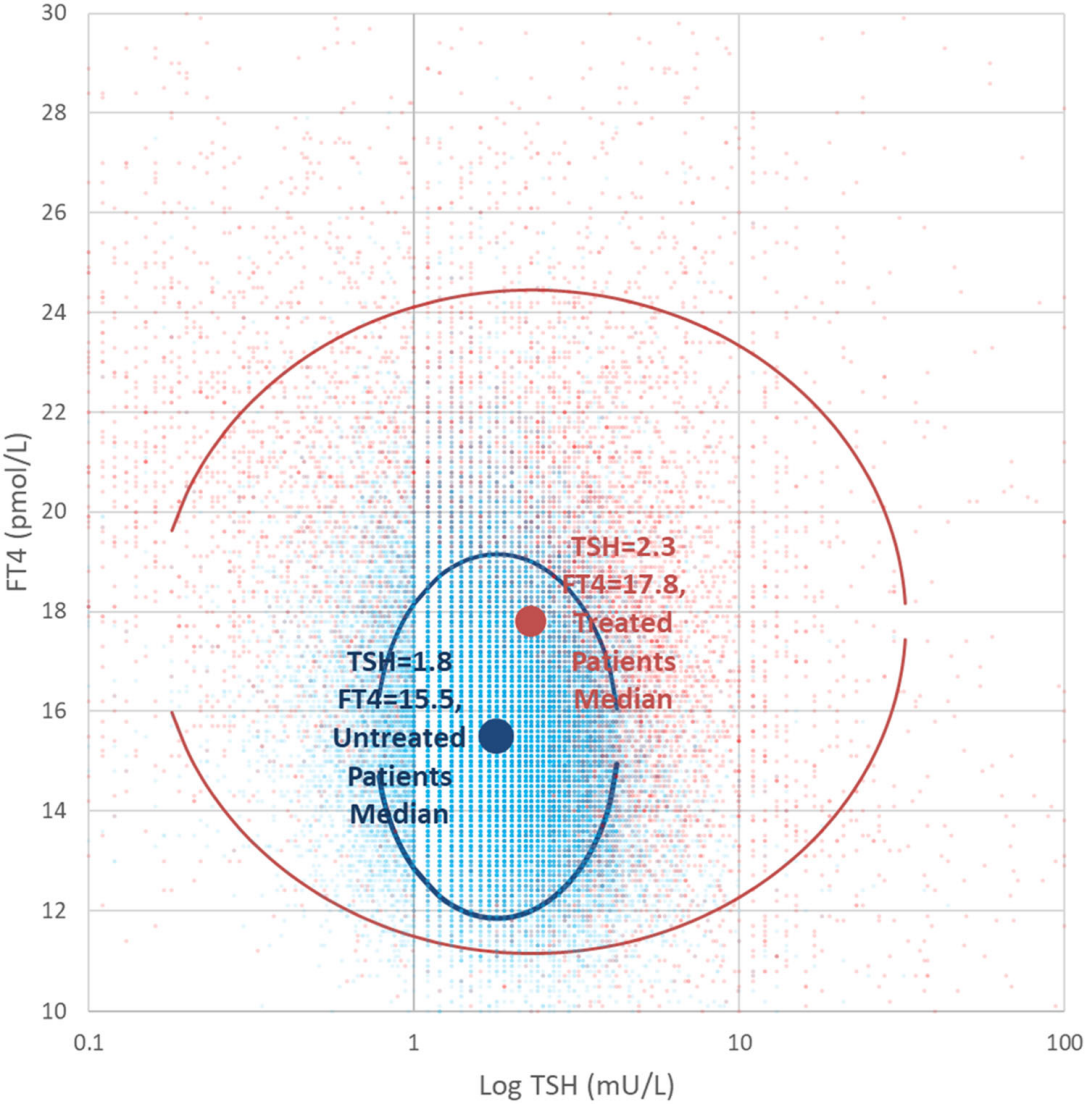
Shows FT4 value against log(TSH) for (a) results for patients who have been treated with LT4. Where more than one result was available the median result in each year was applied and (b) Untreated patients who had a single diagnostic test result during the period. The ellipses are centred on median values and x, y based on 5%ile and 95%ile values for TSH and FT4.

Cluster analysis showed overall for untreated patients' median values for TSH and FT4 were 1.8 mUnits/L and FT4 = 15.5 pmol/L respectively, with 24% patients falling outside the 5%/95% percentile limit. For treated patients median TSH was = 2.3 mUnits/L (+30% vs. untreated) and FT4 was = 17.8 pmol/L (+15% vs. untreated), with 20% of treated patients falling outside the treated 5%/95% percentile boundaries. In all 68% of treated results fell outside the untreated FT4/TSH boundary ellipse. There were some variations in this result when considered by sex (male 63% and female 70%) and by age (age < 60 67%, age ≥ 60 64%) (Figure 1).

**Figure 2:**
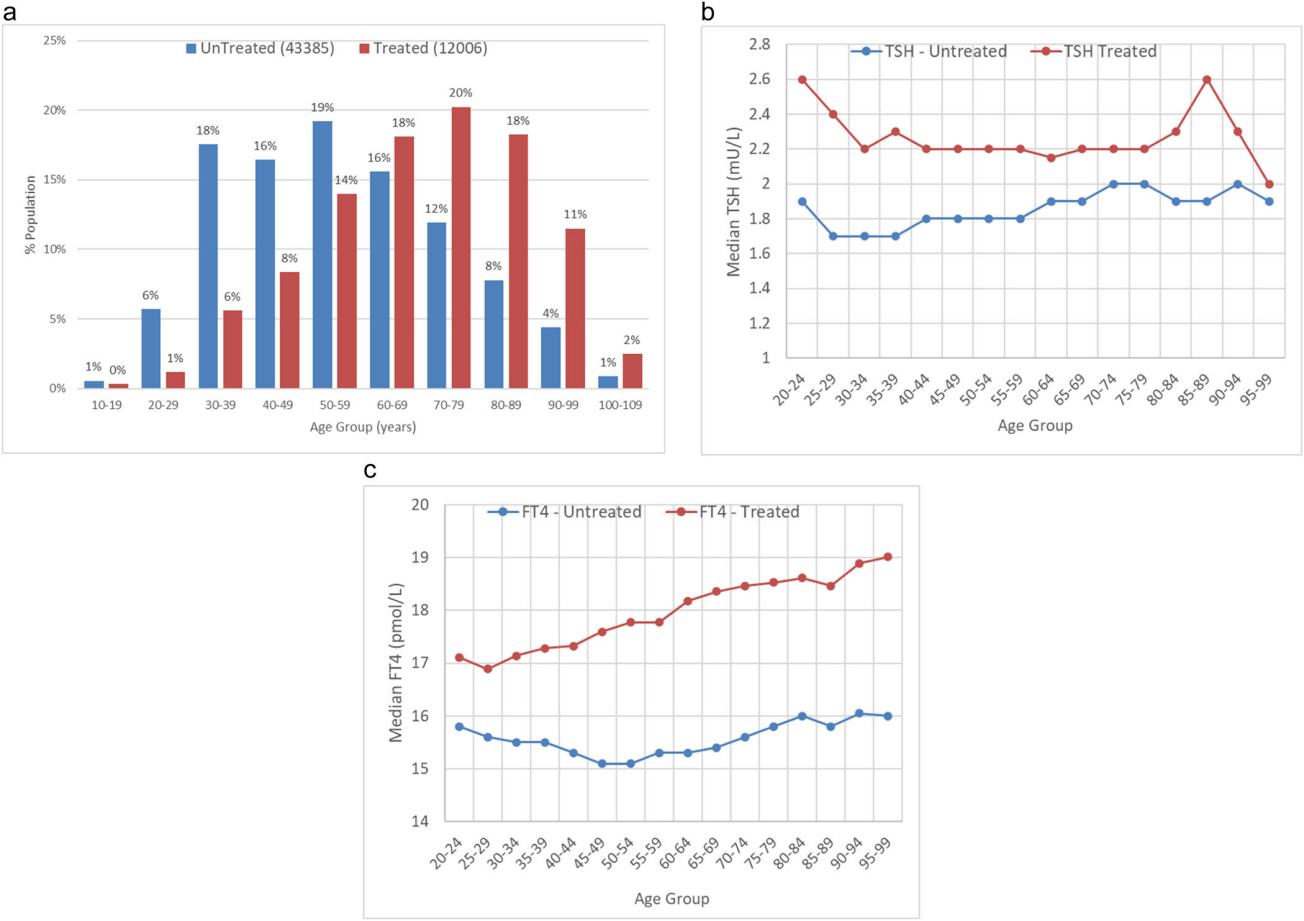
Differences between total treated (monitoring test) and untreated (screening test) populations (a) % total population by age group. (b) Median TSH plotted by age group for treated and untreated patients. (c) Median FT4 by age group.

The age distribution for the treated and untreated individuals was different, as shown in Figure 2a with treated patients being older than those not on LT4 treatment. Variation of TSH and FT4 in the two population was examined:by age.

- Median TSH (Figure 2b) was much higher in the treated population and was relatively unchanged by age, while in the untreated population was lower and increased with age. The difference in TSH between treated and untreated was larger at younger ages and declined with age, to be less at older age,
- Median FT4 (Figure 2c) increased noticeably with age in the treated population, while in the untreated it remained relatively constant. Thus the difference was smaller at younger ages but grew larger with age.

We also applied looked at the relation between treated versus nontreated individuals by gender with an age differential of < 60 years old versus 60 years old or more.

The difference in median values for treated versus nontreated individuals were surprisingly both higher: FT4 by 10% and TSH by 17% with the differences remaining by sex and also in younger or older age groups (Table 1) except for TSH in older women where TSH was the same but FT4 was 15% higher in the treated group.

**Table 1:** Thyroid function test results from blood samples taken in general practices 2008–2012 where patients on levothyroxine were indicated on the request so split between screening (euthyroid) and being treated/monitored (treated) considered by age group and sex.

		Number patients/tests		TSH median (IQR)			FT4 (IQR)			FT4/TSH (IQR)		
Age	Sex	Euthyroid	Treated	Euthyroid	Treated	%Diff	Euthyroid	Treated	%Diff	Euthyroid	Treated	%Diff
Overall		43,394	7019/15,975	1.8 (1.3–2.6)	2.1 (0.9–3.7)	17%	15.5 (14.1–17)	17 (14.8–19.9)	10%	8.5 (5.9–12.4)	8 (4.3–21.6)	−6%
<60	F	18,790	2789/6093	1.8 (1.3–2.5)	2 (0.7–3.8)	11%	15.1 (13.8–16.5)	16.6 (14.3–19.5)	10%	8.5 (5.9–12.3)	8.1 (4.1–24.4)	−5%
<60	M	13,747	925/1699	1.8 (1.3–2.5)	2.2 (1.2–3.8)	22%	15.9 (14.5–17.4)	16.4 (14.3–18.7)	3%	8.8 (6.3–12.5)	7.5 (4.1–14.3)	−15%
≥60	F	5596	2559/6258	2 (1.3–2.8)	2 (0.7–3.5)	0%	15.6 (14.2–17.3)	17.9 (15.6–20.6)	15%	8 (5.4–12.7)	8.8 (4.7–26.6)	10%
≥60	M	5261	917/1925	1.9 (1.3–2.7)	2.5 (1.3–4)	32%	15.6 (14.1–17.1)	16.6 (14.6–19.1)	6%	8.1 (5.6–11.9)	6.5 (3.9–13.5)	−20%

*Note:* On restricting the untreated population to a TSH of 0.14–0.4 mu/L, although around 6% of results were excluded, the overall difference to the total set in final values was very small ‐ the TSH 75%ile = 2.4 was reduced by 8% and the FT4/TSH ratio increased by 2%.

Abbreviations: FT4, free thyroxine; IQR, interquartile range; TSH, thyroid‐stimulating hormone.

## 4 Discussion

We have described here results from a large data set suggesting that current treatment regimens of either low or high dose levothyroxine are not delivering the expected laboratory TFT profiles, with significant numbers of treated patients being well outside the expected values—both TSH and FT4 being significantly higher. This effect seems to be more prevalent in women than men, which is more concerning given the higher number of women requiring treatment. There was a steady increase in TSH by age in the untreated population which was not seen in LT4 treated individuals. Conversely FT4 remained relatively stable with age in untreated individuals but increased with age in LT4 treated individuals.

A better understanding of the relation between thyroid hormone replacement therapy and actual levels of TSH, FT3, and FT4 in patients could allow more tailored and effective therapy for patients with hypothyroidism. We can only speculate as to the reasons for these findings. It may be the case that in some individuals the conversion of thyroxine to triodothyronine at the hypothalamic level is less efficient due to lower deiodinase enzyme activity [19], hence for a given level of circulating FT4 the TSH is higher. Another factor is that it is possible that some patients miss doses and then double or triple the dose prior having their TFTs checked thus resulting in a higher FT4 on the day while TSH remains slightly high in relation to the lag in TSH response to circulating thyroid hormone levels [20].

We believe that these data could help inform decisions about whether LT3 and LT4 combination therapy [21] or NDT [22] could be used more widely to reduce the number of patients with persistent symptoms of hypothyroidism while on treatment.

Gullo et al. [23] described a similar phenomenon regarding higher FT4 in treated versus nontreated individuals. However, for that group, the selection was restricted to those with TSH 0.4–4.0 mu/L and to people who had undergone a total thyroidectomy (athyreotic).

It should be pointed out that the age related increase in TSH has previously been described in the NHANES study in a key paper published in 2002 [24]. In the same paper the authors pointed out that a large proportion of the US population at that time unknowingly have laboratory evidence of thyroid disease.

For the treated hypothyroid individuals, timing of LT4 dose may also be a factor as described by Jansen et al. in 2023 [25]. The authors concluded that the observed FT4 concentrations above the upper limit of the laboratory reference range together with not (completely) suppressed TSH were mainly related to a combination of timing of blood withdrawal and the timing of LT4 intake [25].

We accept that there are significant limitations to the laboratory data set as we do not have information about actual thyroid condition diagnosis, nor do have information as to the type, dose or duration of thyroid hormone replacement. Ethnicity was not available to us. Nevertheless, we have been able to analyse a larger number of laboratory results, and we are confident that we have adequately differentiated screening of untreated individuals from monitoring of patients treated with thyroid hormone replacement.

In conclusion, a better understanding of the relationship between thyroid hormone replacement therapy and actual levels of TSH, FT3, and FT4 in patients vs the physiological equipoise, could allow more tailored and effective therapy for patients with hypothyroidism.

## Author Contributions

Mike Stedman and Adrian H. Heald wrote the manuscript with data analysis by Mike Stedman. Lakdasa D. Premawardhana, Onyebuchi E. Okosieme, Peter N. Taylor, Anthony A. Fryer, and Colin M. Dayan contributed to and have approved the final version of the manuscript. Colin M. Dayan, Lakdasa D. Premawardhana, and Adam Baker provided senior review.

## Conflicts of Interest

All authors declare no conflicts of interest.

## Data Availability

The data that supports the findings of the study are available on reasonable request.
